# Long-Term Outcomes in Stroke Patients with Cognitive Impairment: A Population-Based Study

**DOI:** 10.3390/geriatrics5020032

**Published:** 2020-05-18

**Authors:** Majed Obaid, Clare Flach, Iain Marshall, Charles D. A. Wolfe, Abdel Douiri

**Affiliations:** 1 School of Population Health and Environmental Sciences, Division of Health and Social Care Research, King’s College London, Addison House, Guy’s Campus, London SE1 3QD, UK; clare.flach@kcl.ac.uk (C.F.); iain.marshall@kcl.ac.uk (I.M.); charles.wolfe@kcl.ac.uk (C.D.A.W.); abdel.douiri@kcl.ac.uk (A.D.); 2 Department of Community Medicine and Medical Care for Pilgrims, Faculty of Medicine, Umm Al-Qura University, Makkah 24231, Saudi Arabia; 3 National Institute for Health Research Comprehensive Biomedical Research Centre, Guy’s and St. Thomas’ NHS Foundation Trust, London SE1 9RS, UK

**Keywords:** mild cognitive impairment, vascular dementia, post-stroke, recovery and outcomes

## Abstract

This study assesses five year outcomes of patients with cognitive deficits within the first three months after stroke. Population-based data from the South London Stroke Register between 1995 and 2018 were studied. Cognitive function was assessed using the Abbreviated-Mental-Test or Mini-Mental-State-Examination. Multivariable Poisson regression models with robust standard errors were constructed, to evaluate relative risks (RRs) and associations between post-stroke deterioration in cognitive function during the first three months on dependency, mortality, depression and institutionalisation. A total of 6504 patients with first-ever strokes were registered with a mean age of 73 (SD: 13.2). During the first three months post-stoke, approximately one-third of these stroke survivors either cognitively improved (37%), deteriorated (30%) or remained unchanged (33%). Post-stroke cognitive impairment was associated with increases, in five years, of the risks of mortality, dependency, depression and being institutionalised by RRs 30% (95% confidence interval: 1.1–1.5), 90% (1.3–2.6), 60% (1.1–2.4) and 50% (1.1–2.3), respectively. Deterioration in cognitive function by 10% or more between seven days and three months was associated with an approximate two-fold increased risk in mortality, dependency, and being institutionalised after one year, compared to stable cognitive function; RRs 80% (1.1–3.0), 70% (1.2–2.4) and two-fold (1.3–3.2), respectively. Monitoring further change to maintain cognitive abilities should be a focus to improve outcomes.

## 1. Introduction

Stroke continues to be a major public health concern, affecting more than 10 million individuals every year around the world [1]. Approximately one in four patients dies within one month of stroke onset [2], with half of the survivors becoming dependent on others for daily activities [3]. Advances in public health and medicine have led to a consistent reduction in stroke mortality [4]. As a consequence of the reduced mortality rate, researchers are increasingly paying closer attention to disability after stroke. Research confirms that stroke could result in a cognitive deficit; however, this condition is likely to be ignored and covered by severe physical disability [5].

Cardiovascular disease is the second most common cause of cognitive impairment [6]. It is estimated that around a quarter of individuals have cognitive impairment at three months after their first stroke [7,8] and remain at this level for up to 10 years [7]. Historically, the condition of dementia after stroke was identified by researchers as a vascular dementia. However, not all post-stroke patients who suffer from the cognitive impairment meet the criteria of dementia. Consequently, the term “vascular cognitive impairment” (VCI) superseded the term “vascular dementia” [5]. The degree of cognitive decline of other cognition-impaired patients failing to meet the dementia criteria, could be measured by general screening tools assessing cognitive function. Tools such as the Mini-Mental State Examination (MMSE) score, Montreal Cognitive Assessment Scale (MoCA) score and the Abbreviated Mental Test (AMT) could be utilised.

A recent priority-setting exercise by the James Lind Alliance identified post-stroke cognitive impairment as the leading issue to be investigated due to its influences on the quality of life after stroke of survivors [9,10]. Cross-sectional studies have investigated the association between cognitive impairment and other difficulties, for example disability and limitations in executive function. Significant cognitive impairment has been shown to be associated with disability, dependency, competency and handicap [8,11]. A small number of cohort studies have looked at the long-term outcomes of individuals with post-stroke cognitive impairment other than dementia [12]. Patel’s analysis [13] of the South London Stroke Register found that cognitive impairment was associated with disability and mortality up to four years after the initial stroke; however, too few cohort cases were studied. Heruti [14] found that initial cognitive impairment was indicative of rehabilitation outcomes in the short term. A review of the literature on cognitive impairment highlights the importance of considering functional recovery, neuropsychological impairments and economic burden [12]. However, limited data are available to assess outcomes in a longitudinal study design.

The aims of this study are to determine how post-stroke cognitive impairment and change in cognitive function between stroke onset and three months after stroke are associated with long-term stroke outcomes. The outcomes considered in this study are functional dependency, mortality, depression and being institutionalised in a hospital or care home, up to five years after stroke onset.

## 2. Materials and Methods

### 2.1. Study Population 

We have conducted the study using data from the South London Stroke Register (SLSR). SLSR is a prospective population-based stroke register that was set up in January 1995 and recorded all first-ever strokes with diverse ethnic backgrounds of all ages for an inner-area of South London, including 22 electoral wards in Lambeth and Southwark. Patients who had a first-ever stroke are identified in an area defined by postcodes of Lambeth and Southwark, South London. The total population was 271,817 in 2001 and 357,308 in 2011 according to census data from the Office for National statistics. The latest census data also represents that the population within the study area consists of 56% white, 25% black (14% black African, 7% black Caribbean and 4% other black) and 18% of other ethnic backgrounds [15]. In this study, we used data collected between January 1995 and December 2018. Individuals who agreed to join the register completed a baseline interview and were followed-up at three months and annually thereafter up to five years.

### 2.2. Case Ascertainment

Standardised criteria were applied to ensure completeness of case ascertainment, including multiple notification sources. All patients with a suspected diagnosis of stroke or transient ischemic attack documented in different hospital- and community-based information sources were investigated further for inclusion eligibility. Completeness of case ascertainment has been estimated at 88% by a multinomial-logit capture–recapture model using the methods provided elsewhere [16,17].

### 2.3. Data Collection

The data collected from hospital surveillance of admissions for stroke includes three teaching hospitals (Guy’s, St. Thomas’ and King’s) that cover the study area. In addition, the data also collected from community surveillance of stroke include patients within the study area population who are under the care of all general practitioners (GPs) within the area boundaries. Data were collected by specially trained study nurses and field workers whenever feasible prospectively. A study clinician verified the diagnosis of stroke. On the initial stroke, data were collected on patient demographics, stroke severity, pathological stroke subtypes, treatments before and at stroke onset, cognitive function, disability along with demographic variables and past-medical history (either self-reported or from medical notes) including smoking, hypertension, diabetes mellitus, atrial fibrillation and myocardial diseases (Supplementary File). At follow-up visits, information was collected on disability, dependency, quality of life, cognitive function, depression, recent health events, e.g., stroke recurrence, myocardial infarction. Death data were linked to the Office for National Statistics. 

Stroke was defined based on the World Health Organisation criteria [18]. Pathological stroke subtypes were identified as ischaemic and haemorrhagic. The Glasgow Coma Score (GCS) [19], dichotomised to <13 or ≥13, was used to estimate stroke severity of stroke onset. The data registration used for this study has collected the National Institute of Health Stroke Score (NIHSS) [20] since 2001. However, when the SLSR was initiated at 1995, the decision on stroke severity was based on clinical findings such as GCS and other acute impairments. Having complete NIHSS data since 1995, would have been ideal especially in the analysis of outcomes of cognitive impairment after stroke. However, the variables selected for stroke severity to construct models in this study were chosen for their clinical relevance and their prognostic value, which was observed in two previous studies [21,22].

In this analysis, we were interested in several outcomes covering physical (dependency), mortality (death), mental (depression) and economic factors (institutionalisation). Disability (functional dependency) was measured using the modified Barthel index [23], range 0–20 with scores over 14 indicative of independence. Notification of deaths was provided by linkage to the National Health Service (NHS) digital, who provide the date and cause of death for all individuals on the register. The Hospital Anxiety and Depression Score (HADS) [24] was used to assess depression, each individual achieved a score of 0–21, depression is defined as a score >7 [24]. An individual was recorded as institutionalised if it was reported that they were living in a residential home, nursing home or hospital at the time of follow-up. 

The measure of cognitive impairment recorded in the SLSR has changed over the years. Before 1 January 2000, the Mini-Mental State Exam (MMSE) [25] with values <24 was indicative of impairment. After 1 January 2000, the Abbreviated Memory Test (AMT) [26] was used, where an individual was considered cognitively impaired if they scored <8 [26]. We considered cognitive impairment measured at seven days post-stroke, at three months and a change in score of 10% between these time points as predictors of outcomes. 

When describing cognition function up to three months after stroke, we utilised the full cohort of all individuals able to complete cognitive impairment tests. Our population for the study of non-mortality outcomes was restricted to those who had follow-up at one year and five years post-stroke and were alive at that time. For mortality, all individuals who completed cognitive impairment tests at seven days and at three months were utilised. In addition, we considered information collected on patient demographics, risk factors and stroke pathological subtypes as possible confounders of the association between cognitive impairment and outcomes.

### 2.4. Statistical Analysis 

Baseline characteristics were presented by cognitive impairment category at seven days and at three months after the index of stroke, using descriptive analysis measures; mean and standard deviation or frequencies and percentages as appropriate at both seven days and three months after stroke. Binary outcome measures were tested for crude associations with cognitive impairment using chi-squared tests. Multivariable Poisson regression models with robust standard error of each outcome (mortality, dependency, depression and being institutionalised) on cognitive impairment were constructed in a complete case analysis, to determine relative risk (RR) of each outcome at one year and at five years. We constructed several models of each outcome, considering cognitive impairment at seven days, at three months and the change between seven days and three months as predictors of interest. Change in cognitive impairment at three months after stroke index was calculated on the continuous scale (MMSE/AMT) and defined as a 10% decrease or improvement and remained unchanged from the measurement at seven days. We used a change of 10% of the maximum score as the minimum clinically significant level of change in cognitive function as reported in the previous study [27].

Multivariable models were adjusted for year of stroke, age, sex, ethnicity, socioeconomic status, stroke subtype, previous diagnoses of transient ischemic attack, hypertension, diabetes mellitus, myocardial infarction, atrial fibrillation, smoking status, stroke severity measures (GCS), motor deficit, vascular risk factors, baseline neuropsychological or physical problems, dementia prior to stroke and stroke recurrence. 

To assess the robustness of the results, we performed additional sensitivity analyses of missing data using multiple imputation based on inverse probability weights presented elsewhere [28]. The probability of response was estimated using multivariable logistic regression, including factors associated with dropping out (cognitive score at previous visits, socioeconomic status, age and race). This approach showed a little effect on the estimates. The observed data analysis was therefore used for the present study. Statistical analyses were performed using Stata (2016, StataCorp LP, College Station, TX, USA).

### 2.5. Ethics

Informed written consent and assent, when appropriate, were obtained from all patients or from a next of kin for the individuals who were too impaired to provide written consent. Ethical approval for the study was obtained from the ethics committees of Guy’s and St. Thomas’ Hospital Trust, The King’s College Hospital Research Ethics Committee, Queens Square, Westminster Hospital (London, UK) and The Wandsworth Local Research Ethics Committee. Ethics committee reference number: 01-195, version no.: 1 and dated 30 May 2007.

## 3. Results

### 3.1. Participants’ Characteristics 

A total of 6504 individuals were recorded with a first-ever stroke and agreed to be part of the SLSR between January 1995 and December 2018. Of these, n = 3411 (52%) patients had cognitive function measured at seven days, of them (n = 1204; 35%) had cognitive impairment. A total of n = 1891 patients had cognitive function measured at both seven days and three months. Of them, n = 1608 (85%) completed a follow-up interview at one year, and n = 846 (58%) completed a follow-up interview at five years. A total of n = 2171 (33%) individuals died within three months. Another n = 2000 (30%) and n = 399 (11%) individuals did not have cognitive function measured on stroke onset or three-months after stroke, due to medical reasons. At stroke onset, the medical reasons were communication impairment (n = 992; 49.6%) and coma (n = 737; 37%). The remaining number was due to late registration or because their date of follow-up was not reached (n = 271; 13.4%). A total of n = 31 patients had stroke recurrence by three months after the first stroke; n = 78 patients had stroke recurrence at one year; and n = 112 at five years. Post-stroke cognitive impairment at both seven days and three months was significantly associated with being older >74 years, female (n = 638; 52%), manual worker (n = 680; 56%) and having a more severe stroke, motor deficit, urinary incontinence and being diagnosed with atrial fibrillation. The majority of stroke patients were not on either warfarin or aspirin prior to stroke onset. Characteristics of these patients included sociodemographic, past medical history, case mix, stroke subtypes and year of stroke as presented in Table 1.

### 3.2. Long-Term Stroke Outcomes by the Presence of Cognitive Impairment after Stroke

Cognitive impairment at seven days post-stroke was associated with an increased risk of each of the outcomes considered: mortality, physical dependency, depression and being institutionalised at both one year (Table 2), RRs 80% (1.4–2.3), 90% (1.4–2.7), 40% (1.0–2.0) and two-fold (1.6–2.8); and five years after stroke (Table 3), RRs 30% (1.1–1.5), 90% (1.3–2.6), 60% (1.1–2.4) and 50% (1.1–2.3), respectively. A sensitivity analysis considering cognitive impairment at three months post-stroke presented approximately the same findings (Table S1).

### 3.3. Long-Term Stroke Outcomes by Early Change in Cognitive Function after Stroke

Of the 1891 individuals who were tested for cognitive function between seven days and three months, n = 708 (37%) showed about 10% improvement at three months, an additional n = 569 (30%) showed 10% deterioration, with n = 614 (33%) showing no changes. Table 4 shows that those who had any improvement in cognitive function between seven days and three months were more likely to be younger <65 years old, male (n = 380; 54%), manual worker (n = 439; 62%), from the white ethnic group (n = 474; 67%) and have ischaemic stroke with no previous diagnosis of diabetes. Improvement was greater in those whose first stroke was in 1995–1999 than in those who had theirs in later years. 

Maintaining the same level of cognition between seven days and three months was associated with a reduced risk of dependency, mortality and being institutionalised at one year compared to a deterioration in cognitive function at this early stage (Table 5). A small deterioration by (10%) in cognitive function between seven days and three months was associated with an increased risk of mortality, dependency and being institutionalised at one year compared to maintaining the same level of cognitive function; RRs 80% (1.1–3.0), 70% (1.2–2.4) and two-fold (1.3–3.2), respectively. There was little difference between remaining stable or improving on these outcomes. Details on the findings at one year and five years are shown in Table 5 and Table S2, respectively.

## 4. Discussion

The community-based data from a large and diverse population of South London showed that over the first three months after stroke, approximately one in three stroke survivors, who completed the initial cognitive function assessment, either improved, deteriorated, or stayed the same. The frequency of post-stroke cognitive impairment was comparable to that in previous studies, which was that about a third of stroke survivors developed cognitive impairment after stroke. The presence of cognitive impairment after stroke was associated with a worsening of long-term stroke outcomes including mortality, depression, dependency and being institutionalised. Moreover, early deterioration in cognitive function within the first three months after stroke was strongly associated with negative stroke outcomes, including survival, dependency and being institutionalised, compared to those who had stable cognitive function. These findings build on the hypotheses of those studies that established a cross-sectional association between cognitive impairment and functioning [8,11]. However, the current study provides, for the first time, data on associations in a longitudinal design; updating these findings using a larger population and, also for the first time, evaluating the impact of early decline in cognitive function on stroke outcomes for up to five years.

A previous study showed that stroke survivors with dementia were more often discharged to nursing homes and were at higher risk of three months mortality compared to stroke survivors with no dementia (*p* < 001) [29]. However, the intention of the current study was not to examine dementia but to examine global cognitive impairment after stroke in a large cohort of stroke cases, using MMSE/AMT as a general screening indicator. This study supports the concept of “cognitive impairment no dementia”, which is becoming increasingly relevant in terms of analysis in the field of population health and epidemiology [30]. Our findings showed that the presence of cognitive impairment after stroke is prevalent and seems to increase risk of mortality and physical dependency approximately two-fold; this is similar to findings observed by Patel et al. (2002) [13]. Case-fatality rate among cognitively impaired stroke patients versus cognitively intact patients was reported at one, three and four years after stroke as 14% versus 4%, 34% versus 22% and 50% versus 30%, respectively.

Depression has been reported in stroke patients (n = 143) to be predicted by early post-stroke cognitive impairment, up to six months of follow-up; OR 3.4 (1.2–9.7) [31]. In this study, similar findings were observed over a longer period and with a larger cohort of cases. Stroke itself increases the risk of depression [32]; survivors after stroke present a unique challenge to detect this depression. Stroke-related neurological symptoms such as aphasia, diminished motivation or severe reduction in emotional expressiveness may hinder healthcare practitioners to identify post-stroke depression [33]. Studies have suggested the plausibility of an inverse association, through which cognitive impairment could be predicted by early post-stroke depression. Both conditions seem to entangle and lead to an increase in mortality [34] and utilisation of healthcare services [32]. Findings from a clinical trial were promising, showing that early remission of depressive symptoms after stroke among patients (n = 17) led to an improvement in cognitive function measured by MMSE, from 23.3 (SD = 4.2), at stroke onset, improved to 26.6 (SD = 3.5) at three months follow-up and remained approximately the same for up to two years [35].

There are some possible explanations for the observed poor outcomes [13,36]. First, cardiovascular risk factors such as atrial fibrillation and hypertension tend to be more prevalent across stroke patients with cognitive impairment compared to those among cognitively intact patients. Therefore, cognitive impairment may serve as a replacement for those factors that lead to the onset of more severe stroke, higher risk of stroke recurrence and death. Second, patients with cognitive impairment are more likely to miss medical appointments and regular medications; this could result in less-effective application of secondary stroke preventions and subsequently result in more risk of stroke recurrence, death and disability. Third, cognitive impairment prevents stroke patients from engaging with family, friends, social activities and rehabilitation programmes. Therefore, it subsequently reduces their ability to cope with physical disability and increases the possibility of social isolation and depression.

The present study supports the conclusion that cognitive function improvement after stroke is possible [37,38]. The frequency of cognitive improvement in this study was promising. One in three patients had improvement in cognitive function during the first three months. The observed improvement seems to be a natural outcome, related to a younger age group, male, manual worker, from the white ethnic group, with the absence of diabetes. It was reported that the greatest improvement might happen in the first three months after stroke [39], however, there are factors reducing the chance of progressing such as diabetes [37], cardiovascular and neurodegenerative diseases [38].

Deterioration in cognitive function during the first three months after stroke increases the risk of poor long-term outcomes, compared to a maintenance in the level of cognition with no further deterioration. Many risk factors are suggested to be associated with cognitive deterioration including polypharmacy and previous cognitive decline [40]. We have observed a fluctuating trend of cognitive function over 10 years after stroke with no specific pattern related to age [16]. The challenge now is how to prevent further decline in cognitive function at early stage of stroke onset [41]. Stroke survivors with cognitive impairment might require early detection, continuous screening, comprehensive monitoring, more therapy and longer neurorehabilitation as early as possible after stroke onset [42]. The latest Sentinel Stroke National Audit Programme (SSNAP) annual report indicates that screening of cognitive problems in the first six weeks has improved, reaching 93% in 2018/2019 from 91% in 2016/17. This is very promising progress, indicating that there is an awareness of cognitive issues [43]. However, only 5% of stroke survivors in hospital are considered to be eligible for a review by a psychologist. The first SSNAP annual report for 2013/14 found that only 60% of those found to need support for mood and cognition received it, though it is not reported whether this has improved along with screening [44]. 

Moreover, the Care Quality Commission also highlights that there is a lack of access to rehabilitation for cognitive difficulties in their review of stroke services. They report that neuropsychology for cognitive difficulties was only available to everyone in 35% of primary care trusts (PCTs), and in over 40% of PCTs it was not available at all [45]. Given that deterioration in cognitive function in the first few months after stroke is a strong indicator of poor recovery and that this is the time when individuals are most accessible and have the most contact with health services, the current study confirms that it is possible to improve provision for cognitive difficulties at this time to prevent deterioration. 

This study cannot determine whether those that had a deterioration in cognitive impairment had received less therapeutic treatment than those that did not deteriorate, as we do not have good information collected on therapeutic treatments received. Deterioration in cognitive function itself may not be a direct cause of dependency or death but may lead to other problems. For example, with remembering medication, appointments and the ability to carry out everyday activities that could then impact these outcomes. 

The strengths of our data are capturing a population-based stroke register over a longer period, with a diverse ethnic group, and providing an insight into the overall burden related to post-stroke cognitive impairment. However, this is an observational study and such findings are likely to be subject to unmeasured confounding and attrition bias due to stroke severity, though we have adjusted for all possible confounders and factors associated with missing data. Furthermore, the psychometric screening tools used in this study may underestimate the impact of cognitive impairments, particularly mild cognitive impairment and executive function [46]. Although these are limitations in detecting mild cases, this study has shown poor outcomes related to decline of cognitive function. Due to the observed limitations of this study, it is important to highlight that the cognitive impairment investigated in the present study was that identified after stroke and was not necessarily solely stroke-related cognitive deterioration. Thus, caution must be exercised while interpreting the findings of this study. Similar large, long-term studies of post-stroke cognitive function using sensitive tools to detect all cases, including mild cognitive impairments, will be of benefit to reconfirm the burden of this condition. 

## 5. Conclusions

This population-based study has shown that cognitive impairment is one of the indicators of the long-term impact of stroke. Post-stroke cognitive impairment may improve or deteriorate over time. Our findings showed that during the first three months after stroke, approximately one third of stroke survivors either improved, deteriorated or remained the same. Early deterioration in cognitive function is strongly associated with poor stroke outcomes. The findings highlight that stroke patients should not be lost to healthcare providers especially during the first three months, as close monitoring to maintain cognitive abilities should be a focus to improve stroke outcomes.

## Figures and Tables

**Table 1:** Cognitive function assessment at stroke index and at three months after stroke.

		Seven Days			Three Months		
Variable	Category	Impaired n (%)	Intact n (%)	*p*-Value *	Impaired n (%)	Intact n (%)	*p*-Value *
Total		1204 (35)	2207 (65)		728 (29)	1786 (71)	
Year of first stroke	1995–1999	344 (28)	290 (13)		247 (34)	406 (23)	
	2000–2004	219 (18)	483 (21)		158 (21)	367 (20)	
	2005–2009	395 (32)	584 (26)		136 (18)	386 (21)	
	2010–2014	139 (11)	498 (22)		74 (10)	339(19)	
	2015–2018	107 (9)	352 (16)	<0.001	113 (15)	288 (16)	<0.001
Age group	<65 years	259 (21)	963 (43)		174 (24)	736 (41)	
	65–74 years	303 (25)	541(24)		194 (27)	476 (27)	
	75–84 years	386 (32)	529 (23)		228 (31)	445 (25)	
	85+ years	256 (21)	174 (8)	<0.001	132 (18)	129 (7)	<0.001
Sex	Female	638 (52)	924 (41)	<0.001	380 (52)	778 (43)	<0.001
Ethnicity	White	827 (69)	1457 (66)		425 (58)	1215 (68)	
	Black	314 (26)	625 (28)		250 (34)	464 (26)	
	Other	57 (5)	107 (5)		50 (7)	91 (5)	
	Missing	6 (0.5)	18 (0.8)	0.36	3 (0.4)	16 (0.9)	<0.001
Socioeconomic group	Non-manual	254 (21)	755 (34)		162 (22)	584 (32)	
	Manual	680 (56)	1049 (47)		428 (56)	880 (49)	
	Unknown	187 (15)	326 (15)		89 (12)	243 (14)	
	Missing	81 (7)	74 (3)	<0.001	49 (7)	79 (4)	<0.001
Stroke subtype	Infarct	989 (82)	1888 (85)		618 (84)	1498 (83)	
	Haemorrhagic	185 (15)	273 (12)		99 (14)	249 (13)	
	Unknow	30 (2)	46 (2)	0.3	11 (1)	39 (2)	0.5
Risk factors							
Transient ischaemic attack	No	1037 (86)	1954 (88)		609 (83)	1552 (86)	
	Yes	151 (12)	230 (10)		103 (14)	195 (11)	
	Missing	1 (0.08)	5 (0.2)	0.1	0	2 (0.1)	0.1
Hypertension	No	363 (30)	763 (34)		211 (29)	589 (32)	
	Yes	831 (69)	1430 (64)		502 (68)	1164 (65)	
	Missing	2 (0.1)	2 (0.09)	0.06	1 (0.1)	0	0.1
Diabetes mellitus	No	908 (75)	1730 (78)		532 (73)	1397 (78)	
	Yes	284 (23)	641 (20)		183 (25)	352 (20)	
	Unknown	12 (1)	16 (1)	0.1	13 (2)	37 (2)	0.01
Myocardial infarction	No	1042 (86)	1984 (89)		629 (86)	1565 (87)	
	Yes	138 (11)	190 (8)		80 (11)	171 (10)	
	Missing	24 (2)	33 (2)	0.01	0	1 (0.04)	0.6
Atrial fibrillation	No	943 (78)	1915 (86)		580 (80)	1514 (84)	
	Yes	242 (20)	263 (11)		129 (18)	228 (13)	
	Missing	2 (0.1)	6 (0.2)	<0.001	1 (0.1)	2 (0.1)	0.01
Smoker or ex-smoker	Never	455 (37)	798 (36)		293 (40)	629 (35)	
	Ex-smoker	367 (30)	729 (33)		227 (31)	559 (31)	
	Current smoker	318 (26)	653 (29)		186 (25)	568 (31)	
	Unknown	62 (5)	25 (1)	<0.001	22 (3)	28 (1)	0.002
Stroke severity (case-mix)							
Glasgow coma scale	Severe (<8)	101 (8)	32 (1)		30 (4)	37 (2)	
	Moderate (9–12)	174 (14)	64 (3)		89 (12)	97 (5)	
	Mild (13–15)	928 (77)	2111 (96)	<0.001	609 (84)	1652 (92)	<0.001
Motor deficit	No	231 (19)	658 (30)		155 (21)	511 (28)	
	Yes	948 (78)	1464 (66)		554 (76)	1199 (67)	
	Missing	25 (2)	84 (4)	<0.001	16 (2)	68 (4)	<0.001
Incontinence	No	554 (46)	1816 (82)		402 (55)	1408 (78)	
	Yes	622 (51)	333 (15)		301 (41)	332 (18)	
	Unknown	23 (2)	51 (2)	<0.001	23 (3)	41 (2)	<0.001
Medications prior to stroke							
Warfarin	No	1076 (89)	2012 (91)		617 (84)	1547 (86)	
	Yes	50 (4)	71 (3)		24 (3)	60 (3)	
	Unknown	58 (5)	63 (2)		37 (5)	67 (4)	
	Missing	20 (1)	61 (2)	0.002	50 (6)	112 (6)	0.437
Aspirin	No	798 (66)	1520 (68)		453 (62)	1184 (66)	
	Yes	328 (27)	563 (25)		188 (26)	423 (24)	
	Unknown	58 (5)	63 (3)		37 (5)	67 (4)	
	Missing	20 (1)	61 (3)	0.003	50 (6)	112 (6)	0.188
Stroke recurrence							
Stroke recurrence (up to 1 year)	No	1188 (99)	2167 (98)		716 (98)	1755 (98)	
	Yes	14 (1)	35 (2)	0.320	12 (2)	30 (2)	0.814
Stroke recurrence (up to 5 years)	No	1184 (98)	2147 (97)		712 (98)	1732 (97)	
	Yes	18 (2)	55 (3)		15 (2)	50 (3)	
	Missing	0 (0)	1 (0.06)	0.054	1 (0.1)	4 (0.2)	0.516

* *p*-value < 0.05 for chi-squared test for comparison between cognitively impaired and intact groups.

**Table 2:** One year outcomes by seven day cognitive impairment.

Cognitive Function at 7 Days	Outcome at 1 Year		N	Adjusted * RR	*p*-Value
Death	No	Yes	3411		
Intact	2024 (71%)	183 (34%)			
Impaired	845 (29%)	359 (66%)		1.8 (1.4–2.3)	<0.001
Physical dependency	Independent	Dependent	1938		
Intact	1160 (77%)	182 (43%)			
Impaired	356 (23%)	240 (57%)		1.9 (1.4–2.7) ^†^	<0.001
Depression	Normal	Borderline/Depressed	1562		
Intact	866 (80%)	321 (66%)			
Impaired	210 (20%)	165 (34%)		1.4 (1.0–2.0) ^‡^	0.01
Institutionalised	No	Yes	1964		
Intact	1240 (75%)	125 (42%)			
Impaired	424 (25%)	175 (58%)		2.1 (1.6–2.8)	<0.001

* Adjusted for year of stroke; sociodemographic factors; stroke type; vascular risk factors; smoking status; stroke severity measures; medications prior to stroke, recurrence of stroke and prior to stroke dementia. RR—relative risk. ^†^ Additionally adjusted for baseline disability. ^‡^ Additionally adjusted for baseline depression.

**Table 3:** Five year outcomes by seven day cognitive impairment.

Cognitive Function at 7 Days	Outcome at 5 Years		N	Adjusted * RR	*p*-Value
Death	No	Yes	3411		
Intact	1636 (75%)	571 (46%)			
Impaired	521 (24%)	683 (54%)		1.3 (1.1–1.5)	<0.001
Physical dependency	Independent	Dependent	1022		
Intact	649 (80%)	116 (55%)			
Impaired	162 (20%)	95 (45%)		1.9 (1.3–2.6) ^†^	<0.001
Depression	Normal	Borderline/Depressed	941		
Intact	512 (82%)	220 (70%)			
Impaired	116 (18%)	93 (30%)		1.6 (1.1–2.4) ^‡^	0.021
Institutionalised	No	Yes	1057		
Intact	696 (77%)	93 (575)			
Impaired	197 (22%)	71 (43%)		1.5 (1.1–2.3)	0.02

* Adjusted for year of stroke; sociodemographic factors; stroke type; vascular risk factors; smoking status; stroke severity measures; medications prior to stroke, recurrence of stroke and prior to stroke dementia. ^†^ Additionally adjusted for baseline disability. ^‡^ Additionally adjusted for baseline depression.

**Table 4:** Baseline measures by change in cognitive impairment between seven days and three months.

Variable	Category	Deteriorated n (%)	Stable n (%)	Improved n (%)	*p*-Value *
Total		569 (30)	614 (33)	708 (37)	
Year of 1st stroke	1995–1999	100 (18)	83 (14)	269 (38)	
	2000–2004	175 (31)	145 (24)	86 (12)	
	2005–2009	122 (21)	136 (22)	167 (24)	
	2010–2014	91 (16)	124 (20)	106 (15)	
	2015–2018	81 (14)	126 (21)	80 (11)	<0.001
Age group	<65 years	201 (35)	246 (40)	220 (31)	
	65–74 years	140 (25)	158 (26)	204 (29)	
	75–84 years	167 (29)	162 (26)	204 (29)	
	85+ years	61 (11)	48 (8)	80 (11)	0.018
Sex	Male	288 (51)	363 (59)	380 (54)	
	Female	281 (49)	251 (41)	328 (46)	0.011
Ethnicity	White	344 (60)	431 (70)	474 (67)	
	Black	188 (33)	155 (25)	202 (29)	
	Other	36 (6)	25 (4)	31 (4)	
	Unknown	1 (0.2)	3 (0.4)	1 (0.1)	0.014
Socioeconomic group	Non-manual	155 (27)	215 (35)	187 (26)	
	Manual	330 (58)	276 (45)	439 (62)	
	Unknown	68 (12)	101 (16)	64 (9)	
	Missing	16 (3)	22 (4)	18 (3)	<0.001
Stroke subtype	Infarct	491 (86)	525 (85)	588 (83)	
	Haemorrhagic	67 (12)	77 (13)	110 (16)	
	Unknown	11 (2)	12 (2)	10 (1)	0.285
Transient ischaemic attack	No	495 (87)	539 (88)	619 (87)	
	Yes	72 (13)	69 (11)	85 (12)	
	Missing	0	2 (0.3)	0	0.517
Hypertension	No	179 (31)	229 (37)	189 (27)	
	Yes	386 (68)	384 (63)	518 (73)	
	Missing	1 (0.1)	0	0	0.001
Diabetes mellitus	No	422 (74)	491 (80)	552 (77)	
	Yes	146 (25)	119 (19)	149 (21)	
	Missing	1 (0.2)	4 (1)	7 (1)	0.036
Myocardial infarction	No	510 (90)	542 (88)	628 (89)	
	Yes	55 (10)	63 (10)	69 (10)	
	Missing	4 (1)	9 (2)	11 (2)	0.687
Atrial fibrillation	No	488 (86)	525 (85)	607 (86)	
	Yes	77 (14)	83 (14)	93 (13)	
	Missing	2 (0.3)	0	0	0.308
Smoker or ex-smoker	Never	227 (40)	224 (36)	256 (36)	
	Ex-smoker	171 (30)	228 (37)	224 (31)	
	Current	168 (30)	158 (25)	224 (32)	
	Missing	0	0	1 (0.1)	0.132
Glasgow coma score	Severe (<8)	2 (1)	18 (1)	11 (4)	
	Moderate (9–12)	4 (3)	63 (4)	29 (12)	
	Mild (13–15)	148 (96)	1408 (94)	208 (83)	<0.001
Motor deficit	No	142 (25)	188 (31)	184 (26)	
	Yes	413 (72)	405 (66)	511 (72)	
	Missing	14 (2)	21 (3)	13 (2)	0.045

* *p*-value < 0.05 for chi-squared test for comparison between cognitively impaired and intact groups.

**Table 5:** One year outcomes by change in cognitive function between seven days and three months.

Change in Cognitive Function	Outcome at 1 Year		N	Adjusted * RR	*p*-Value
Death	No	Yes	1891		
Stable	588 (33%)	26 (24%)			
Deteriorated	517 (29%)	52 (47%)		1.8 (1.1–3.0)	0.017
Improved	676 (38%)	32 (29%)		0.6 (0.3–1.2)	0.227
Physical dependency	Independent	Dependent	1366		
Stable	389 (36%)	58 (21%)			
Deteriorated	299 (27%)	94 (35%)		1.7 (1.2–2.4) ^†^	0.03
Improved	407 (37%)	119 (44%)		0.9 (0.8–1.3) ^†^	0.850
Depression	Normal	Borderline/Depressed	1092		
Stable	299 (39%)	86 (27%)			
Deteriorated	224 (29%)	99 (31%)		1.3 (0.8–2.4) ^‡^	0.292
Improved	251 (32%)	133 (42%)		1.3 (0.7–2.2) ^‡^	0.336
Institutionalised	No	Yes	1384		
Stable	420 (35%)	33 (17%)			
Deteriorated	332 (27%)	65 (34%)		2.0 (1.3–3.2)	0.001
Improved	442 (37%)	92 (48%)		1.1(0.7–1.7)	0.655
The reference group is the stable group					

* Adjusted for 7 day cognitive impairment, year of stroke; sociodemographic factors; stroke type; vascular risk factors; smoking status; stroke severity measures; medications prior to stroke and recurrence of stroke. ^†^ Additionally adjusted for baseline disability. ^‡^ Additionally adjusted for baseline depression.

## Supplementary Materials

The following are available online at https://www.mdpi.com/2308-3417/5/2/32/s1, Table S1: Long-term outcomes by three-month cognitive impairment, Table S2: Five-year outcomes by change in cognitive function between seven-days and three-months.
