# Supplementary material for: Long-Term Outcomes in Stroke Patients with Cognitive Impairment: A Population-Based Study
Source: Geriatrics (Basel). 2020 May 18;5(2):32. doi: 10.3390/geriatrics5020032 (PMC7345015; doi:10.3390/geriatrics5020032)
Supplement: Supplementary file 1 [file geriatrics-05-00032-s001.pdf]

Comorbidities:

Data collected on past medical history of each stroke patients admitted to hospital either self-reported or from hospital and primary care records. It includes a medical history of vascular risk factors; hypertension, diabetes mellitus, atrial fibrillation; disability (BI); past medical history of cognitive impairment or depression. It also considers pharmacological treatment prior to or at the time of the stroke including antiplatelet agents excluding aspirin (dipyridamole, or clopidogrel), aspirin, other than warfarin anticoagulants (heparin, novel or direct oral anticoagulant), warfarin. For this study, the use of these variables is considered with acknowledgment of that past medical history may not always be accurately, complete or systematically recorded in medical records. However, most of the data of past medical history used in this thesis is relevant to stroke management, and this likely improve its completeness and accuracy in medical records. Data on smoking was collected using validated questions. Patients are categorised as current smoker, ex-smoker or never smoker. If feasible, amount of tobacco smoked per day and the duration of being smokers for, are also recorded.

Supplemental material

Table S1. Long-term outcomes by three-month cognitive impairment

Cognitive impairment at 3 months	Outcome at 1 year			Outcome at 5 years		
	N	Adjusted* RR	p-value	N	Adjusted* RR	p-value
Death	2,514	2.7 (1.7-4.2)	<0.001	2514	2.03 (1.54-2.68)	<0.001
Physical dependency	1,797	4.7 (3.3-6.5) †	<0.001	880	2.30 (1.38-3.82) †	0.001
Depression	1391	2.1 (1.4-3.1) ‡	<0.001	794	1.27 (0.79-2.04) ‡	0.316
Institutionalised	1,813	3.7 (2.6-5.4)	<0.001	909	2.20 (1.33-3.63)	0.002

*adjusted for: year of stroke; socio-demographic factors; stroke type; vascular risk factors; smoking status; stroke severity measures; medications prior to stroke, recurrence of stroke and prior to stroke dementia.
† additionally adjusted for baseline disability.
‡ additionally adjusted for baseline depression.

Table S2. Five-year outcomes by change in cognitive function between seven-days and three-months.

Change in cognitive function	Outcome at 5 years		N	Adjusted* RR	p-value
Death	No	Yes	1891		
Stable	447 (35%)	137 (24%)			
Deteriorated	385 (29%)	184 (33%)		1.3 (1.0-1.6)	0.05
Improved	471 (35%)	237 (42%)		1.0 (0.8-1.3)	0.788

Physical dependency	Independent	Dependent	680		
Stable	199 (36%)	33 (24%)			
Deteriorated	154 (28%)	46 (34%)		1.5 (0.9-2.4) †	0.123
Improved	192 (35%)	56 (41%)		1.5 (0.7-3.6) †	0.27
Depression	Normal	Borderline/Depressed	626		
Stable	162 (38%)	63 (31%)			
Deteriorated	125 (29%)	59 (29%)		1.1 (0.7-1.5) ‡	0.783
Improved	137 (32%)	80 (39%)		1.0 (0.6-1.5) ‡	0.950
Institutionalised	No	Yes	703		
Stable	204 (35%)	34 (29%)			
Deteriorated	176 (30%)	32 (28%)		1.1 (0.6-1.8)	0.732
Improved	209 (35%)	48 (42%)		0.8 (0.5-1.5)	0.861
The reference group is the stable group					

*adjusted for: 7-day cognitive impairment, year of stroke; socio-demographic factors; stroke type; vascular risk factors; smoking status; stroke severity measures; medications prior to stroke and recurrence of stroke.

†additionally adjusted for baseline disability.

‡additionally adjusted for baseline depression.
